# Effect of Bushen Huoxue Prescription on Cognitive Dysfunction of KK-Ay Type 2 Diabetic Mice

**DOI:** 10.1155/2021/6656362

**Published:** 2021-03-12

**Authors:** Shao-Yang Zhao, Huan-Huan Zhao, Ting-Ting Hao, Wei-Wei Li, Hao- Guo

**Affiliations:** ^1^Institute of Basic Medical Sciences, Xiyuan Hospital, China Academy of Chinese Medical Sciences, Beijing 100091, China; ^2^Research Studio of Integration of Traditional and Western Medicine, First Hospital, Peking University, Beijing 100034, China; ^3^Nutrition Department, LinYi People's Hospital, Linyi 276000, China

## Abstract

Diabetic cognitive impairment is one of the common complications of type 2 diabetes, which can cause neurological and microvascular damage in the brain. Bushen Huoxue prescription (BSHX), a compound Chinese medicine, has been used clinically to treat diabetes-induced cognitive impairment. However, its underlying mechanisms remain unclear. In this study, KK-Ay diabetic model mouse was administered BSHX daily for 12 weeks. Bodyweight, random blood glucose (RBG), and fasting blood glucose (FBG) were measured every 4 weeks. Triglycerides (TG), cholesterol (TC), high-density lipoprotein cholesterol (HDL-C), low-density lipoprotein cholesterol (LDL-C), fasting serum insulin (FINS), and Morris water maze were tested after 12 weeks of administration. On the day of sacrifice, the hippocampus was collected for pathological staining and advanced glycation end products (AGEs) analysis to evaluate the neuroprotective effect of BSHX. Our results showed that BSHX treatment significantly ameliorated the T2DM related insults, including the increased bodyweight, blood glucose, TG, insulin levels, AGEs, the reduced HDL-C, the impaired spatial memory, and the neurological impairment. Moreover, Western blot analysis showed that increased expression of receptors of AGEs (RAGEs), inducible nitric oxide synthase (iNOS), cyclooxygenase-2 (COX-2), and activation of nuclear factor-*κ*B (NF-*κ*B) in the hippocampus were significantly inhibited by BSHX treatment. These results indicate that BSHX can significantly ameliorate glucose and lipid metabolism dysfunction, reduce the morphological changes in hippocampus tissues, and improve the cognitive function of KK-Ay mice. These protective effects of BSHX may involve regulation of the AGEs/RAGE/NF-*κ*B signaling pathway.

## 1. Introduction

Type 2 diabetes mellitus (T2DM) is a metabolic disease mainly characterized by long-term hyperglycemia, which is affected by multiple factors [1]. According to a survey, 693 million people worldwide will suffer from T2DM by 2045 [2]. During the occurrence and development of this disease, the metabolism of carbohydrates, lipids, and proteins is abnormal, resulting in long-term damage to various tissues such as the eye, kidney, nerve, cardiocerebral vessel, and eventually functional impairment and failure. Diabetic cognitive dysfunction is one of the main chronic complications. A foreign epidemiological survey shows that T2DM can increase the risk of cognitive dysfunction by 40% [3, 4]. Diabetes has become an independent risk factor for cognitive dysfunction [5]. Diabetes combined with cognitive dysfunction can eventually result in memory disorder, cognitive dysfunction, aphasia, and apraxia and bring heavy burden to society [6–11]. Therefore, the prevention and treatment of diabetic cognitive dysfunction is of great significance to improve the prognosis and quality of life of T2DM patients.

According to traditional Chinese medicine, the core pathogenesis of diabetic cognitive dysfunction is kidney deficiency and blood stasis. Bushen Huoxue prescription (BSHX), a newly formulated compound Chinese medicine, is to add leech on the basis of modified Wuzi Yanzong prescription (Table 1) to increase the function of promoting blood circulation and dredging collaterals on the basis of tonifying kidney. Its clinical application has proven that BSHX can significantly alleviate memory decline in the patients in addition to its hypoglycemic effect [12–15]. Previous studies have reported that some traditional Chinese medicines or monomers in this prescription have good glucose-controlling and neuroprotective effects, in which Chinese wolfberry can not only reduce blood glucose but also improve cognitive impairment in T2DM mice [16–19]. Icariside can improve cerebral ischemia-reperfusion-induced microcirculatory disturbance, improve blood flow, and reduce neuron loss and apoptosis in the CA1 area of the hippocampus in *Gerbillinae* [15], while hyperoside and schisandrin *B* can inhibit the neurotoxicity of amyloid *β*-protein (A*β*) by inhibiting the activation of the mitochondrial apoptosis pathway [19, 20]. However, the improvement effect of BSHX on diabetic cognitive dysfunction still lacks systematic pharmacodynamic evaluation. The aim of the present study was to investigate the effect of BSHX on diabetic cognitive impairment in a spontaneous T2DM animal model: KK-Ay mice.

## 2. Materials and Methods

### 2.1. Materials

BSHX was purchased from Beijing Kangrentang Pharmaceutical Co., Ltd. (Beijing, China). The medicinal components of BSHX are listed in Table 1. Protamine zinc recombinant human insulin was from Eli Lilly and Company (Indiana, USA). Metformin was purchased from Shanghai Squibb Pharmaceutical Co., Ltd. (Shanghai, China). High-fat diet for mice was obtained from Beijing HFK Bioscience Co., Ltd. (Beijing, China). Normal diet for mice was obtained from Beijing Keao Xieli Feed Co., Ltd. (Beijing, China). Stroke-physiological saline solution was from Beijing Double-Crane Pharmaceutical Co., Ltd. (Beijing, China). Mouse ultrasensitive insulin ELISA kit was from ALPCO (USA). TUNEL kit was purchased from Roche Group (Basel, Switzerland). Triglyceride assay kit (GPO-PAP) and total cholesterol assay kit (COD-PAP) were obtained from BioSino Biotechnology and Science Inc. (Beijing, China). High-density lipoprotein cholesterol (HDL-C) and low-density lipoprotein cholesterol (LDL-C) assay kits were purchased from Nanjing Jiancheng Co., Ltd. (Nanjing, China). AGEs ELISA kit was purchased from Andy Gene Biotechnology Co., Ltd. (Beijing, China). Nuclear extraction kit was obtained from KeyGEN Biotech Inc. (Nanjing, China). Primary antibodies for RAGE, iNOS, COX-2, p-NF-*κ*B, NF-*κ*B, and rabbit IgG were obtained from Cell Signaling Technology (Boston, MA, USA).

### 2.2. HPLC Analysis

Referring to the study of our research group [21], BSHX extracts were analyzed using a Shimadzu prominence liquid chromatography platform (Kyoto, Japan) equipped with two LC-20AT pumps, a CTO-20A column oven, a DGU-20A5R degasser, an SIL-20A autosampler, and an SPD20AD detector. Chromatographic separation was conducted on a AichromBond-AQ C18 column (250 mm × 4.6 mm, 5 *μ*m; Abel Industries Ltd., Canada), protected by a Phenomenex® C18 guard cartridge (3 × 4 mm, 5 *μ*m; Torrance, CA, USA). The mobile phase consisted of ACN (A) and 0.1% aqueous formic acid (B) and was delivered at 1.0 mL/min with the following gradient program: 0–35 min, 0–3% B; 35–100 min, 3–22% B; 100–115 min, 22–35% B; 115–130 min, 35–35% B; 130–140 min, 35–100% B; 140–145 min, 40–100% B; and 145–155 min, 100–100% B. The column was maintained at 40°C. At the end of each run, the delivery of 100% A was performed for another 14 min for system reequilibration. The monitor wavelength was set at 235 nm, 254 nm, and 280 nm, respectively.

### 2.3. Animal Grouping and Drug Administration

All animal experiments complied with the European Union Guidelines (2010/63/EU) and were performed as per the Guidelines of the Animal Research Committee of Peking University after being approved by the Institutional Animal Care and Use Committee of Peking University First Hospital.

Twelve-week-old male KK-Ay mice (*N* = 50), weighing 30–35 g, were purchased from Beijing HFK Bioscience Co., Ltd. (license no. SCXK (Jing) 2016–0005). Twelve-week-old male C57BL/6J mice (*N* = 10), weighing 23–25 g, were purchased from Beijing Vital River Laboratory Animal Technology Co., Ltd. (License No. SCXK (Jing) 2016–0001). KK-Ay mice were fed with high-fat diet, and C57BL/6J mice were fed with general diet. All animals were kept in the animal room of Xiyuan Hospital, China Academy of Chinese Medical Sciences. The mice could drink water freely and were reared for 10 days under the conditions of 22 ± 1°C, 40% ± 5% humidity, and a 12 h light cycle. Blood glucose was measured by the glucose oxidase method. Only KK-Ay mice satisfying the requirement of random blood glucose (RBG) ≥11.1 mmol/L or fasting blood glucose (FBG) ≥7.0 mmol/L were taken as DM samples.

The mice were randomly divided into six groups as follows: (1) control (untreated C57BL/6J mice); (2) model (untreated KK-Ay mice); (3) low BSHX (KK-Ay mice treated with 0.5 g/kg BSHX); (4) medium BSHX (KK-Ay mice treated with 1 g/kg BSHX); (5) high BSHX (KK-Ay mice treated with 2 g/kg BSHX); and (6) metformin (KK-Ay mice treated with 250 mg/kg metformin). All drugs were given orally, 0.1 mL/10g each day for 12 weeks. The control group and model group were given the equivalent volume of distilled water in the same manner for contrast.

### 2.4. Oral Glucose Tolerance Test (OGTT)

Blood was taken from caudal veins of mice fasted for 8 hours for FBG measuring. After giving mice 2 g/kg glucose solution orally, the blood glucose levels at the timepoints of 30 min (BG30), 60 min (BG60), and 120 min (BG120) were measured, and the curve was drawn. The area under the curve (AUC) was then calculated according to the formula AUCg = (FBG + BG30) × 0.5 ÷ 2 + (BG30 + BG60) × 0.5 ÷ 2 + (BG60 + BG120) × 1 ÷ 2.

### 2.5. Insulin Tolerance Test (ITT)

Blood was taken from caudal veins of mice fasted for 8 hours for FBG detection. The blood glucose levels of mice were detected at 30 min (BG30), 60 min (BG60), and 120 min (BG120) after 0.26 IU/kg insulin was injected intraperitoneally, and the curve was drawn. The AUC was computed by AUCg = (FBG + BG30) × 0.5 ÷ 2 + (BG30 + BG60) × 0.5 ÷ 2 + (BG60 + BG120) × 1 ÷ 2.

### 2.6. Fasting Serum Insulin (FINS) and the Blood Lipid Test

Concentrations of FINS in blood taken from caudal veins of mice fasted for 8 hours were tested using commercial ELISA kits following the manufacturer's protocol. Concentrations of total triglycerides (TG) and total cholesterol (TC) in serum were measured using triglyceride assay kits (GPO-PAP) and total cholesterol assay kits (COD-PAP), respectively. High-density lipoprotein cholesterol (HDL-C) and low-density lipoprotein cholesterol (LDL-C) in serum were detected with corresponding assay kits according to manufacturer's protocol.

### 2.7. Morris Water Maze (MWM) Test

After 12 weeks of treatment, spatial and related forms of learning and memory were assessed by the MWM test [22]. Briefly, mice were individually trained in a circular pool (120 cm in diameter and 50 cm in height), which was filled with 30 cm deep water at a constant temperature of 25 ± 1°C. There was a platform (9 cm in diameter) at the center of each of four quadrants of the pool. On each side of the walls of the four quadrants, distinctly colored papers were disposed as a visual positional hint. On the first 2 days, each mouse was subjected to visible platform training, in which the platform was submerged 1 cm beneath the water surface and was indicated by a flag. On days 3–5, hidden platform training was carried out with the flags removed for cultivating the spatial learning and memory retention ability of mice to find the platform.

The escape latency of mice in finding the platform was then tested for 5 days. Briefly, four trials were performed on each mouse per day with about 1 h interval in between. During each trial, mice were released into the water facing the pool wall from one of the 4 starting positions and allowed to locate the submerged platform for a maximum of 120 s. Mice failing to find the platform within 120 s were gently guided to the platform and allowed to stay on it for 15 s. On day 6, the platform was removed, and a probe trial was performed to record the time spent in the target quadrant where the escape platform was placed and the number of crossings over the original position of the platform.

### 2.8. Hematoxylin and Eosin (H&E) Staining

Normal saline was infused into mice under anesthesia via the left ventricle, followed by injection of 4% paraformaldehyde for fixation. After being harvested, brains were kept in 10% modified formaldehyde fixative, embedded in paraffin, and cut into 4 *μ*m thick slices successively for H&E staining and optical observation of pathological changes.

### 2.9. Nissl Staining

The paraffin sections were dewaxed, debenzoled by gradient alcohol, and washed with distilled water. After being dyed at 37°C for 10 minutes and washed with distilled water for 3 times, the samples were differentiated and decolorized by 95% and 75% ethanol until the background under the microscope was clear. The samples were dehydrated routinely and sealed with transparent and neutral gum prior to observation under an optical microscope.

### 2.10. TUNEL Staining

The paraffin sections were fixed with 4% paraformaldehyde and washed with distilled water before being incubated at 37°C for 20 min with protein digestive enzyme. The TUNEL apoptosis kit was then used for staining as directed.

### 2.11. Measurement of Advanced Glycation End Products (AGEs)

The hippocampal tissues of mice were collected after 12 weeks of treatment. The concentrations of AGEs in hippocampus were detected by ELISA kit (Andy Gene Biotechnology Co., Ltd., Beijing, China) according to the manufacturer's instructions.

### 2.12. Western Blot Analysis

After the anesthetized mice were transcardially perfused with saline through the left ventricle, brains were collected and lysed with RIPA buffer (50 mM Tris-HCl, 300 mM NaCl, 0.5% TritonX-100, 5 mM EDTA, and protease inhibitor cocktail) for 30 min on ice. The cell lysates were centrifuged at 13,000 rpm and 4°C for 15 min before determination of protein concentrations by Bradford assay. Nuclear and cytoplasmic proteins were extracted with the Nuclear Extraction Kit (KeyGEN Biotech Inc., Nanjing, China) according to manufacturer's instructions. Equal amounts of protein were separated with SDS-PAGE and subsequently transferred to polyvinylidene fluoride membranes. The blots were blocked with 5% nonfat milk in PBST and then incubated with primary antibodies at 4°C overnight. Afterward, the blots were washed adequately in PBST and incubated with horseradish peroxidase-labeled goat anti-rabbit secondary antibody at room temperature for 1 h. The washed blots were then developed with the enhanced chemiluminescent substrate and visualized with the Tanon 5200 Imaging Analysis System (Tanon Science & Technology Co. Ltd., Shanghai, China).

### 2.13. Statistical Methods

All data were expressed as mean ± standard deviation (SD). One-way analysis of variance (ANOVA) and Bonferroni's post hoc test were adopted for statistical analysis. *P* < 0.05 was considered statistically significant, and *P* < 0.01 was considered statistically highly significant.

## 3. Results

### 3.1. Identification of the Components of BSHX Extract

**Figure 1:**
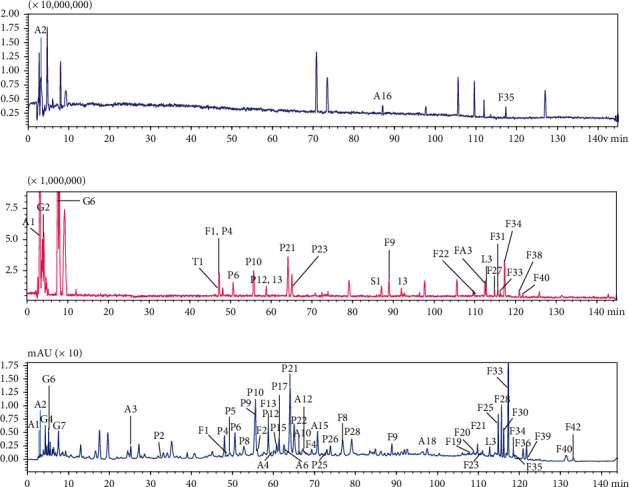
Representative chromatograms analysis of BSHX. (a) Detection in the positive polarity. (b) Detection in the negative polarity. (c) HPLC fingerprint of BSHX with a monitoring wavelength of 280 nm.

Combining application of high-performance liquid chromatography (HPLC) and mass spectrometry (HPLC-MS) to analyze the main components of BSHX extract, a total of 114 compounds were identified, including 43 flavonoids (F1–F43), 28 small molecular phenolic acids (P1–P28), 17 cinnamoyl polyamines (A1–A17), 11 glycolipids (G1–G11), 8 fatty acids (FA1–FA8), 4 lignins (L1–L4), 2 semiterpene glycosides (T1-T2), and 1 saponin compound (S1). LC/ESI-IT-TOF-MS^n^ analysis results showed that the flavonoids in BSHX were mainly derived from *Epimedium*, cinnamoyl polyamines and glycolipids were mainly derived from wolfberry, and small molecular phenolic acids were the main sources in *Plantago* seed, *Cuscuta* seed, and raspberry (Figure 1 and Supplementary Data).

### 3.2. BSHX Helps Prevent KK-Ay Mice from Gaining Bodyweight

**Figure 2:**
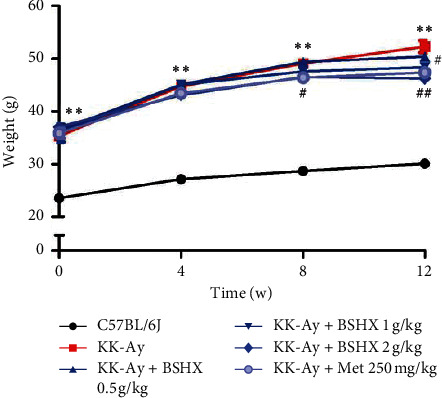
Weight changes of mice in each group after 12 weeks of administration.

As shown in Table 2, untreated KK-Ay mice gained significantly more bodyweight than C57BL/6J mice from the beginning (*P* < 0.01 vs. the C57BL/6J group), and they developed obesity at the 12th week. The weight growth of KK-Ay mice fed by BSHX was slower than that of untreated KK-Ay mice (Figure 2). There were statistical differences regarding the bodyweight between the KK-Ay mice fed by 2 g/kg BSHX and the untreated KK-Ay mice after 8 weeks of treatment (*P* < 0.05 vs. the KK-Ay group). Twelve weeks later, the bodyweight of mice treated by different doses of BSHX was statistically different from that of untreated KK-Ay mice (*P* < 0.05 or *P* < 0.01 vs. the KK-Ay group). The bodyweight showed no significant increase and maintained at a stable level in the high BSHX (2 g/kg) group.

Bodyweight of mice in each group was examined every 4 weeks. Data were expressed as mean ± SD (*n* = 10). ^*∗∗*^*P* < 0.01 vs. the C57BL/6J group. ^#^*P* < 0.05, ^##^*P* < 0.01 vs. the KK-Ay group.

### 3.3. BSHX Inhibits the Increase of Random Blood Glucose (RBG)

**Figure 3:**
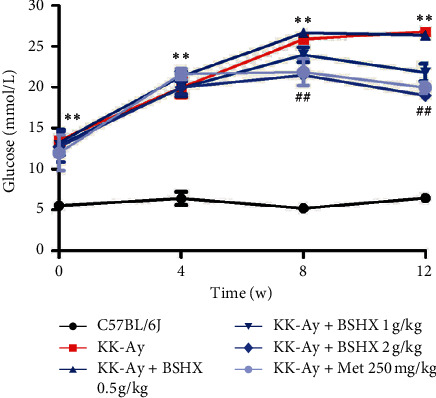
Random blood glucose changes of KK-Ay in each group after 12 weeks of administration.

According to Table 3, the RBG of mice in the model group rose continuously and was significantly different from that in the control group (*P* < 0.01 vs. the C57BL/6J group). Four weeks after gavaging, blood glucose increased significantly in the model group and all treatment groups, but no difference was found between each treatment group and the model group. Eight weeks after treatment, the blood glucose level of mice in the high BSHX (2 g/kg) group was significantly lower than that in the model group (*P* < 0.01 vs. the KK-Ay group). BSHX (2 g/kg) showed the similar effect to metformin in reducing blood glucose. After continuous administration for 12 weeks, blood glucose was notably improved in both medium BSHX (1 g/kg) and high BSHX (2 g/kg) groups (*P* < 0.01 vs. the KK-Ay group), and a high dose of BSHX (2 g/kg) showed a better effect (Figure 3).

Blood samples were collected from mice caudal veins in each group to detect random blood glucose every 4 weeks. Data were expressed as mean ± SD (*n* = 10). ^*∗∗*^*P* < 0.01 vs. the C57BL/6J group. ^##^*P* < 0.01 vs. the KK-Ay group.

### 3.4. BSHX Reduces the Fasting Blood Glucose (FBG) Level

**Figure 4:**
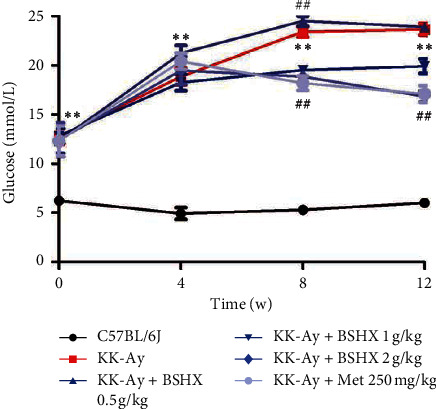
Fasting blood glucose changes of KK-Ay in each group after 12 weeks of administration.

After gavage for 8 weeks, the FBG levels of mice were improved in the metformin group and all BSHX groups, especially in the medium BSHX (1 g/kg) group. After gavage for 12 weeks, the FBG levels of mice in the medium BSHX (1 g/kg) and high BSHX (2 g/kg) groups were significantly improved (*P* < 0.01 vs. KK-Ay group), and a high dose of BSHX (2 g/kg) had a similar effect to metformin in reducing the FBG level (Table 4 and Figure 4).

Blood samples were collected from mice caudal veins in each group to detect fasting blood glucose every 4 weeks. Data are expressed as mean ± SD (*n* = 10). ^*∗∗*^*P* < 0.01 vs. the C57BL/6J group. ^##^*P* < 0.01 vs. the KK-Ay group.

### 3.5. BSHX Ameliorates Glucose Tolerance and Insulin Resistance

**Figure 5:**
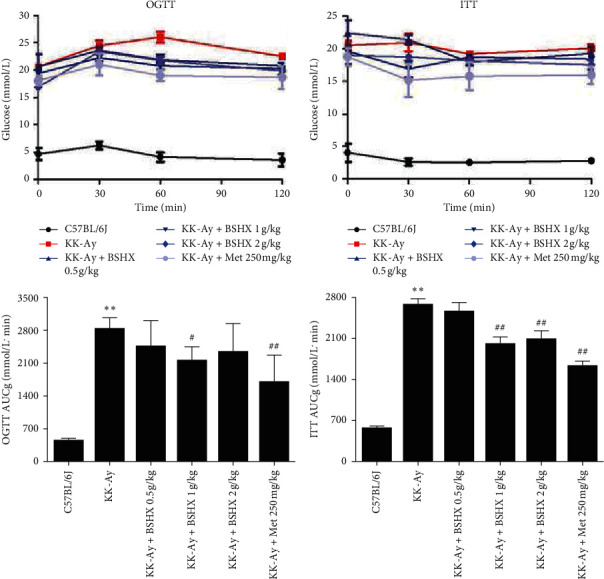
The effect of BSHX on OGTT and ITT. (a) After 12 weeks of administration, glucose solution was given 2 g/kg orally to mice in each group, and the blood glucose curve was drawn at the timepoints of 30 min, 60 min, and 120 min, respectively. (b) After injecting insulin intraperitoneally with a dosage of 0.26 IU/kg to mice in each group, the blood glucose curve was drawn at the timepoints of 30 min, 60 min, and 120 min, respectively. Data are expressed as mean ± SD (*n* = 10). ^*∗∗*^*P* < 0.01 vs. the C57BL/6J group. ^#^*P* < 0.05, ^##^*P* < 0.01 vs. the KK-Ay group.

The oral glucose tolerance of mice in each group was measured after continuous gavage for 12 weeks (Figure 5(a)). Specifically, high glucose solution was given to mice in each group. Within the first 30 min, the blood glucose level of all mice increased, especially the KK-Ay mice in the model group. For the next 30 min, the blood glucose level in all BSHX groups began to decrease, while that of the model group peaked. After 120 min, the blood glucose level tended to stabilize in all groups. The statistical analysis results suggested that the AUC of OGTT in the medium BSHX (1 g/kg) group and the metformin group was significantly lower than that in the model group (*P* < 0.05 or *P* < 0.01 vs. the KK-Ay group). However, compared with the model group, the AUC of low (0.5 g/kg) and high BSHX (2 g/kg) groups decreased but showed no statistical difference.

As indicated in Figure 5(b), the blood glucose level in the KK-Ay model mice presented an increasing trend due to stress hyperglycemia within the first 30 min after insulin injection. In contrast, the blood glucose levels of mice in all BSHX groups and the metformin group decreased in varying degrees within the first 30 min. After insulin injection for 60–120 min, the blood glucose level of mice in all groups gradually decreased and finally maintained unchanged. Moreover, compared with the model group, all BSHX groups, especially the medium BSHX (1 g/kg) group, had a significantly smaller AUC of ITT (*P* < 0.01 vs. the KK-Ay group). The above results demonstrated that the 12-week administration of BSHX (especially with a dose of 1 g/kg) could tremendously ameliorate insulin resistance of mice.

### 3.6. BSHX Downregulates the FINS Level and Promotes Blood Lipid Metabolism

FINS, TC, TG, LDL-C, and HDL-C levels were detected in mice after 12 weeks of gavage.  Table 5 shows that FINS, TC, TG, and LDL-C levels of the KK-Ay mice in the model group were significantly higher than those in the control group, while the HDL-C level was on the contrary (*P* < 0.01 or *P* < 0.001 vs. the C57BL/6J group). Compared with that of the model group, the FINS level was significantly reduced in the metformin group (*P* < 0.01 vs. the KK-Ay group) and decreased in all BSHX groups. For TC, TG, LDL-C, and HDL-C, a high dose of BSHX (2 g/kg) significantly reduced TG and increased HDL-C (*P* < 0.05 vs. the KK-Ay group).

### 3.7. BSHX Improves the Cognitive Function of KK-Ay Mice

**Figure 6:**
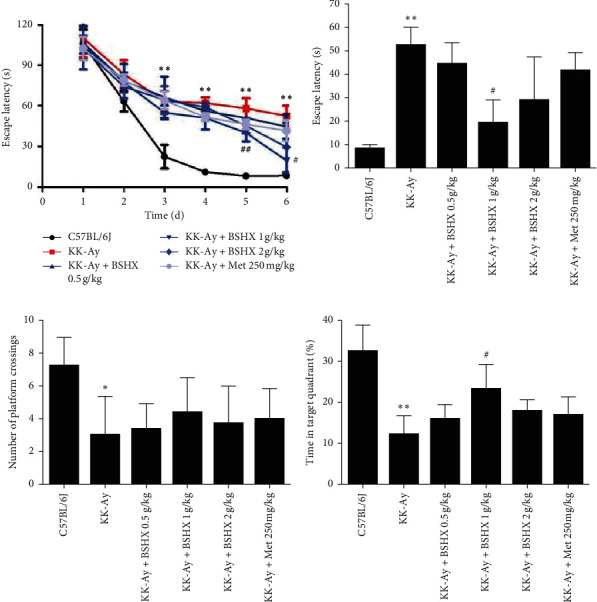
The effect of BSHX on spatial memory of KK-Ay. (a) The mean escape latency time during the training period of mice in each group. Time, seconds. (b) Analysis of escape latency of each group of mice in the formal navigation test. Time, seconds. (c) The number of platform crossings during the spatial probe test. (d) The percent of total time spent in the target of quadrant during the spatial probe test. Data are expressed as mean ± SD (*n* = 10). ^*∗*^*P* < 0.05, ^*∗∗*^*P* < 0.011 vs. the C57BL/6J group. ^#^*P* < 0.05, ^##^*P* < 0.01 vs. the KK-Ay group.

The MWM test was carried out to examine the spatial and related forms of learning and memory of mice. The escape latency gradually decreased in all groups over 5 days of training (Figure 6(a) and Table 6), and the decrease rate was significantly lower in the model group than that in the control group from the third day (*P* < 0.01 vs. the C57BL/6J group). However, compared with the model group, the decline of the escape latency was accelerated in the medium BSHX (1 g/kg) group on day 5 (*P* < 0.01 vs. the KK-Ay group). The escape latency on day 6 further confirmed that BSHX (1 g/kg) significantly improved memory impairment, as displayed in Figure 6(b) and Table 6 (*P* < 0.01 vs. the KK-Ay group). Besides, the escape latency in other BSHX groups also deceased, but the difference between them and the model group was not significantly different.

In the probe trial (Figures 6(c) and 6(d)), a putative measurement of spatial learning and memory retention, there were significantly less platform crossings and a smaller percentage of time spent in the target quadrant in the model group than in the control group (*P* < 0.05 or *P* < 0.01 vs. the C57BL/6J group). The time that mice in the medium BSHX (1 g/kg) group spent in the target quadrant was significantly more than that in the model group (*P* < 0.05 vs. the KK-Ay group). The forementioned two indexes were improved in the other two BSHX groups (Figures 6(c) and 6(d)), but no significant difference was observed.

### 3.8. BSHX Reduces the Morphological Changes in Hippocampus Tissues

**Figure 7:**
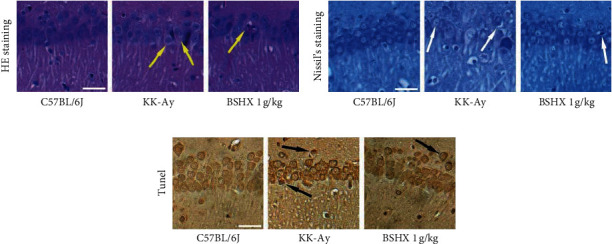
Staining analysis of the effect of BSHX on hippocampal tissue of KK-Ay mice. (a) The results of H&E stain of neurons in hippocampal CA1 region of mice in the C57BL/6J group, KK-Ay group, and KK-Ay + BSHX 1 g/kg group. (b) The results of Nissl stain of neurons in hippocampal CA1 region of mice in each group. (c) The results of TUNEL stain of neurons in hippocampal CA1 region of mice in each group. Scale bar = 50 *μ*m.

Neuronal damage, especially in the hippocampus, is the pathological basis of cognitive dysfunction in DM [23–27]. The staining patterns of CA1 pyramidal cells in the hippocampus of mice in the control, model, and medium BSHX (1 g/kg) groups were measured after the MWM test (Figure 7). The H&E staining results of brain tissues revealed that the hippocampal neurons of the C57BL/6J mice were well-stained and closely arranged with intact structure, while the nuclei of the hippocampal neurons of the KK-Ay model mice were fixed and contracted with enlarged extracellular spaces and disordered arrangement. In the medium BSHX (1 g/kg) group, the neurons in the hippocampus were arranged neatly and stained homogeneously, and neuronal damage was partially recovered (as shown by the yellow arrow). Nissl staining showed that Nissl bodies in the control group were dark blue, and the cells were closely arranged. In the model group, edema appeared around the neurons and the number of cell layers decreased. Besides, Nissl bodies were lightly stained and indistinctly demarcated. In the medium BSHX (1 g/kg) group, no significant edema of neurons was observed, the cells were closely arranged, and Nissl bodies were stained more homogeneously than those in the model group (as shown by the white arrows). In addition, according to the results of TUNEL staining (which could stain apoptotic cells into dark brown), the dark brown color of neurons in the CA1 region of the hippocampus in the model group suggested that many neurons were apoptotic. The neurons in the medium BSHX (1 g/kg) group were more lightly stained than those in the model group, indicating that cell apoptosis was improved (as shown by the black arrows).

### 3.9. BSHX Inhibits the Expression of the AGEs/RAGE/NF-*κ*B Signaling Pathway

**Figure 8:**
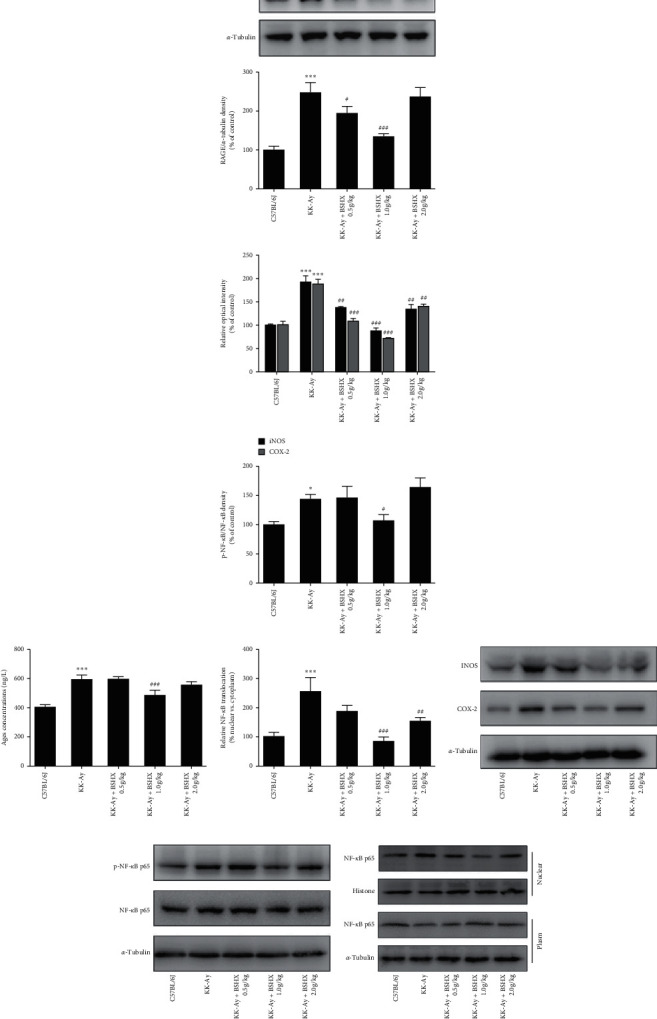
The effect of BSHX on the expression of AGEs, RAGE, iNOS, COX-2, and p-NF-*κ*B. (a) Level of AGEs. (b) Representative Western blots and quantitative analysis of RAGE in each group. (c) Representative Western blots and quantitative analysis of iNOS and COX-2 in each group. (d) Representative Western blots and quantitative analysis of NF-*κ*B and p-NF-*κ*B in each group. (e) Representative Western blot analysis of NF-*κ*B p65 nuclear translocation. Data were expressed as mean ± SD (*n* = 10). ^*∗*^*P* < 0.05, ^*∗∗∗*^*P* < 0.001 vs. the C57BL/6J group. ^#^*P* < 0.05, ^#^*P* < 0.01, ^###^*P* < 0.001 vs. the KK-Ay group.

AGEs play an important role in the pathological development of diabetic cognitive impairment. First, the content of AGEs in the hippocampus of mice was detected by ELISA. According to Figure 8(a), compared with the control group, the model group had significantly increased AGEs content (*P* < 0.001 vs. the C57BL/6J group), and the medium BSHX (1.0 g/kg) group had significantly decreased AGEs content (*P* < 0.01 vs. the KK-Ay group). As for the receptor of AGEs (RAGE), it also dramatically increased in the model group (*P* < 0.01 vs. the C57BL/6J group), but BSHX (0.5 g/kg and 1.0 g/kg) significantly inhibited its expression (*P* < 0.05 or *P* < 0.001 vs. the KK-Ay group) (Figure 8(b)). When AGEs bind to RAGE, they can activate the NF-*κ*B signaling pathway and produce inflammatory factors to destroy the neuron. Therefore, Western blot was adopted to detect whether the NF-*κ*B signaling pathway was activated. As shown in Figure 8(c), iNOS and COX-2, two important inflammatory proteins in the NF-*κ*B signaling pathway, were significantly increased in the brain of the KK-Ay model mice (*P* < 0.001 vs. the C57BL/6J group), while BSHX could notably downregulate the expression of these two proteins (*P* < 0.01 or *P* < 0.001 vs. the KK-Ay group). Meanwhile, a remarkable increase in the phosphorylated NF-*κ*B level was observed in the model group (*P* < 0.05 vs. C57BL/6J group). After 12 weeks of BSHX treatment, the phosphorylated NF-*κ*B level significantly declined (*P* < 0.05 vs. the KK-Ay group) (Figure 8(d)). In addition, the Western blot analysis showed that the model group provoked the translocation of NF-*κ*B p65 from the cytoplasm to the nucleus, and this process was significantly blocked by BSHX (1 g/kg and 2 g/kg) treatment (*P* < 0.01 or *P* < 0.001 vs. the KK-Ay group, Figure 8(e)).

## 4. Discussion

In this study, the spontaneously mutated KK-Ay model mice with T2DM were intervened with drugs, and the effects of BSHX of different doses on the bodyweight, blood glucose and lipid, insulin level, behavioral cognition, and brain morphology of T2DM mice were analyzed. Besides, the pharmacodynamics of BSHX was assessed. The results showed that the bodyweight, blood glucose, TG, TC, LDL-C, and insulin level of T2DM KK-Ay mice in the model group increased significantly, while the HDL-C level was on the contrary. These results suggested that mice in the model group showed hyperglycemia, hyperlipidemia, and insulin resistance. The MWM test also revealed obvious learning and memory disorders in mice aged 25 weeks in the model group. After intragastric administration for 12 weeks, the above symptoms of KK-Ay mice treated with different doses of BSHX or with metformin were alleviated and improved in varying degrees. These results were consistent with the previous related study by our group [28].

However, different doses of BSHX had different effects. BSHX improved bodyweight and blood glucose in a dose-dependent manner. In the high BSHX (2 g/kg) group, the blood glucose level was significantly reduced by the 8th week after gavage. What is more, 2 g/kg of BSHX achieved the same hypoglycemic effect as metformin by the 12th week after gavage. The results of OGTT and ITT were different. After 12 weeks of administration, 1 g/kg of BSHX showed a better performance than 2 g/kg of BSHX in improving the OGTT and ITT of mice. Besides, the FINS level in the medium BSHX (1 g/kg) group was also lower than that in the low (0.5 g/kg) and high BSHX (2 g/kg) groups. However, the metformin was superior to 1 g/kg of BSHX in improving OGTT and ITT, and only the metformin could significantly reduce the insulin level. It suggested that metformin should be preferred in the treatment of T2DM patients in case of no contraindications.

In the aspect of blood lipid and cholesterol, only a high dose of BSHX (2 g/kg) could significantly reduce TG and increase the HDL-C level after 12 weeks of intragastric administration. No significant reduction was found in blood lipid or cholesterol in the low (0.5 g/kg) and medium BSHX (1 g/kg) groups and the metformin group. The reason might be that taking metformin alone was not enough to reduce blood lipid in T2DM mice with a severe condition in the later stage. Moreover, KK-Ay mice were always fed with high-fat feed without diet control, resulting in the sustained high blood lipid level. The high dose of BSHX, however, could lower the lipid level since its concentration of lipid-decreasing components was higher than that in low-dose and medium-dose BSHX. The specific lipid-lowering components required further screening and separation. In terms of learning and memory, the MWM test in the 25th week demonstrated that only the escape latency and stay time in the target quadrant of mice in the medium BSHX (1 g/kg) group were significantly improved, compared with those in the model group. The reason might be that a dose of 2 g/kg was higher than the clinical dosage. The components that originally showed significant efficacy had such a high concentration in the high BSHX group that they produced a negative effect. The specific causes needed to be further analyzed and investigated. The MWM test results reminded us that metformin should be used in combination with other drugs to improve cognitive impairment in T2DM patients. The pathomorphology of the mouse brain was observed through H&E staining, Nissl staining, and TUNEL staining. The results revealed that BSHX could improve the damage of hippocampal neurons in mice.

Neuroinflammatory mechanisms play a crucial role in the development of cognitive dysfunction in T2DM. The anti-inflammatory treatment stems from the “inflammatory theory” of T2DM, in which advanced glycation end products (AGEs) play an important role in the inflammatory response [29–31]. Kalninova et al. detected the levels of inflammatory markers and AGEs in blood of T2DM patients with cognitive impairment and normal people by high-performance liquid chromatography (HPLC) and fluorescence spectrophotometry [32]. The results showed that the levels of AGEs, IL-6, IL-8, TNF-*α*, and monocyte chemotactic protein-1 (MCP-1) in plasma of T2DM patients were significantly higher than those of the normal control group, indicating that there may be a relationship between AGEs and neuroinflammatory factors in T2DM. Advanced glycosylation end products (AGEs), the nonenzymatic glycosylation of proteins or lipids, are found in plasma, microglia, astrocytes, the hippocampus, and the brains of diabetic patients [33]. Therefore, some researchers suspect that AGEs may be involved in neuroinflammation in the brain of type 2 diabetics, leading to diabetic cognitive dysfunction. It has been found that AGEs and AGEs receptor (RAGE) in macrophages can cause oxidative stress and activate NF-*κ*B through the mitogen-activated protein kinase (MAPK) signaling pathway [34, 35]. In addition, AGEs could bind to RAGE and activate the downstream NF-*κ*B signaling pathway, leading to phosphorylation of I*κ*B-*ɑ*. The phosphorylated I*κ*B-*ɑ* is degraded by ubiquitination, and then, the released NF-*κ*B is free in the cytoplasm and quickly transferred to the nucleus, where it specifically binds to the corresponding gene transcription site and induces the expression of inflammation factors, such as cytokines such as interleukin-6 (IL-6) and tumor necrosis factor-*ɑ* (TNF-*ɑ*) [36]. These cytokines could induce the recruitment of adaptive immune system cells into the central nervous system [35]. Wang et al. studied the effect of danshensu on the loss of learning and memory function of T2DM mice mediated by AGEs. The results showed that danshensu could partially block the expression of phosphorylated p38 mitogen-activated protease (p-p38), cyclooxygenase-2 (COX-2), NF-*κ*B, and RAGE and inhibit the increase of TNF-*ɑ*, IL-6, and prostaglandin E2 (PGE2), which indicated that AGEs could mediate the cognitive dysfunction of T2DM mice caused by neuroinflammation. Consistent with the above reports, in this study, we found that BSHX could not only significantly downregulate the expression of AGEs and RAGE but also reduce the levels of phosphorylated NF-*κ*B, nitric oxide synthase (iNOS), and COX-2 in a dose-dependent manner. These results suggested that BSHX could resist neuroinflammation by inhibiting the activation of the AGEs/RAGE/NF-*κ*B signaling pathway. Nevertheless, the specific mechanism required further research given the fact that traditional Chinese medicine compounds often exert their effects though multiple pathways and multiple targets.

## 5. Conclusion

BSHX can significantly ameliorate glucose and lipid metabolism dysfunction, reduce the morphological changes in hippocampus tissues, and improve the cognitive function of KK-Ay mice. The mechanism underlying the above effects of BSHX may be related to the inhibition of activation of the AGEs/RAGE/NF-*κ*B signaling pathway. The results of this study provide a pharmacological basis for the application of BSHX in the treatment of diabetic cognitive dysfunction.

## Figures and Tables

**Table 1:** Composition of BSHX.

Herbal composition	Part used	Percentage of total weight
*Cuscuta chinensis* Lam. (菟丝子)	Fruit	25
*Lycium barbarum* L (枸杞子)	Fruit	25
*Rubus chingii* Hu. (覆盆子)	Fruit	12
*Schisandra chinensis (Turcz.)* Baill.(五味子)	Fruit	3
*Plantago asiatica* L. (车前子)	Seed	6
*Epimedium brevicornu* Maxim. (淫羊藿)	Stem and leaf	25
*Hirudo nipponica* Whitman (水蛭)	The whole body	4

**Table 2:** Bodyweight in each group after 12 weeks of administration (*g*, $\bar{\chi }$ ± *s*, *n* = 10).

	Week 0	Week 4	Week 8	Week 12
C58BL/6J	23.58 ± 0.74	27.16 ± 0.46	28.71 ± 1.7	30.10 ± 1.6
KK-Ay	35.33 ± 1.39^*∗∗*^	44.74 ± 1.39^*∗∗*^	49.13±±1.34^*∗∗*^	52.28 ± 1.11^*∗∗*^
KK-Ay + BSHX 0.5 g/kg	36.41 ± 1.44	45.10 ± 1.14	49.39 ± 1.75	50.38 ± 1.29^*#*^
KK-Ay + BSHX 1 g/kg	35.88 ± 1.63	45.07 ± 1.2	47.58 ± 1.5	48.39 ± 1.26^*#*^
KK-Ay + BSHX 2 g/kg	37.07 ± 1.58	43.8 ± 1.7	46.35 ± 1.23^*#*^	46.28 ± 1.37^*##*^
KK-Ay + Met 250 mg/kg	35.86 ± 1.92	44.34 ± 2.5	46.41 ± 2.3^*#*^	47.39 ± 1.35^*##*^

Data are expressed as mean ± SD (*n* = 10). ^*∗∗*^*P* < 0.01 vs. the C57BL/6J group. ^#^*P* < 0.05, ^##^*P* < 0.01 vs. the KK-Ay group.

**Table 3:** Random blood glucose changes of mice in each group (mmol/L, $\bar{\chi }$ ± *s*, *n* = 10).

	Week 0	Week 4	Week 8	Week 12
C57BL/6J	5.5 ± 0.36	6.39 ± 0.86	5.19 ± 0.31	6.43 ± 0.51
KK-Ay	13.53 ± 0.99^*∗∗*^	19.93 ± 1.19^*∗∗*^	25.87±±0.44^*∗∗*^	26.80 ± 0.47^*∗∗*^
KK-Ay + BSHX 0.5 g/kg	13.27 ± 1.54	21.3 ± 0.64	26.63.39 ± 0.75	26.37 ± 0.76
KK-Ay + BSHX 1 g/kg	13.88 ± 1.63	19.89 ± 1.1	23.97.58 ± 0.9	21.79 ± 1.06^##^
KK-Ay + BSHX 2 g/kg	11.75 ± 1.58	19.17 ± 0.67	21.46 ± 0.51^##^	18.95 ± 0.57^##^
KK-Ay + Met 250 mg/kg	12.36 ± 1.92	21.6 ± 1.24	21.83 ± 1.64^##^	19.93 ± 0.75^##^

Data are expressed as mean ± SD (*n* = 10). ^*∗∗*^*P* < 0.01 vs. the C57BL/6J group. ^##^*P* < 0.01 vs. the KK-Ay group.

**Table 4:** Fasting blood glucose changes of KK-Ay in each group (mmol/L, $\bar{\chi }$ ± *s*, *n* = 10).

	Week 0	Week 4	Week 8	Week 12
C57BL/6J	6.23 ± 0.37	4.93 ± 0.6	5.3 ± 0.41	6 ± 0.36
KK-Ay	12.77 ± 0.78^*∗∗*^	18.87 ± 0.85^*∗∗*^	23.40 ±± 0.5^*∗∗*^	23.67 ± 0.86^*∗∗*^
KK-Ay + BSHX 0.5 g/kg	13.33 ± 1.26	21.23 ± 0.78	19.53.39 ± 0.46^##^	23.11 ± 0.91
KK-Ay + BSHX 1 g/kg	12.15 ± 0.76	18.27 ± 0.85	18.87 ± 0.33^##^	19.93 ± 0.74^##^
KK-Ay + BSHX 2 g/kg	13.43 ± 1.69	19.49 ± 1.43	19.1 ± 0.72^##^	16.84 ± 0.56^##^
KK-Ay + Met 250 mg/kg	11.36 ± 1.52	20.43 ± 0.81	18.2 ± 0.72^##^	17.10 ± 0.81^##^

Data are expressed as mean ± SD (*n* = 10). ^*∗∗*^*P* < 0.01 vs. the C57BL/6J group. ^##^*P* < 0.01 vs. the KK-Ay group.

**Table 5:** Determination of FINS, TC, and TG in each group at week 12 ($\bar{\chi }$ ± *s*, *n* = 10).

	FINS (ng/mL)	TC (mmol/L)	TG (mmol/L)	LDL-C (mmol/L)	HDL-C (mmol/L)
C57BL/6J	1.32 ± 0.67	1.39 ± 0.12	0.57 ± 0.12	0.31 ± 0.06	1.68 ± 0.30
KK-Ay	10.4 ± 2.31^*∗∗*^	3.55 ± 0.63^*∗∗*^	2.16±±0.44^*∗∗*^	0.50 ± 0.08^*∗∗*^	0.75 ± 0.13^*∗∗∗*^
KK-Ay + BSHX 0.5 g/kg	9.25 ± 2.21	2.80 ± 0.88	1.62.39 ± 0.97	0.47 ± 0.09	0.78 ± 0.39
KK-Ay + BSHX 1 g/kg	7.54 ± 1.43	3.07 ± 0.48	1.54 ± 0.41	0.44 ± 0.08	0.73 ± 0.22
KK-Ay + BSHX 2 g/kg	8.58 ± 1.3	2.88 ± 0.85	1.36 ± 0.62^#^	0.45 ± 0.04	1.30 ± 0.16^#^
KK-Ay + Met 250 mg/kg	6.52 ± 1.17^##^	2.74 ± 0.90	1.6 ± 0.36	0.42 ± 0.05	0.80 ± 0.15

Data are expressed as mean ± SD (*n* = 10). ^*∗∗*^*P* < 0.01, ^*∗∗∗*^*P* < 0.001 vs. the C57BL/6J group. ^#^*P* < 0.05, ^##^*P* < 0.01 vs. the KK-Ay group.

**Table 6:** The escape latency of mice in the navigation test ($\bar{\chi }$ ± *s*, *n* = 10).

	Day 1 (s)	Day 2 (s)	Day 3 (s)	Day 4 (s)	Day 5 (s)	Day 6 (s)
C57BL/6J	106 ± 10.5	63.33 ± 7.50	22.33 ± 8.74	11.10 ± 2.6	8.04 ± 1.12	8.33 ± 1.53
KK-Ay	110 ± 14.11	83.35 ± 10.41	63.33±±7.64^*∗∗*^	62.3 ± 4.7^*∗∗*^	58.47 ± 7.55^*∗∗*^	52.67 ± 7.5^*∗∗*^
KK-Ay + BSHX 0.5 g/kg	106.7 ± 12.58	78.16 ± 12.74	66.57 ± 15.18	56.86 ± 3.60	51.23 ± 2.71	44.67 ± 8.96
KK-Ay + BSHX 1 g/kg	105.3 ± 6.66	77.33 ± 7.37	55.01 ± 5.21	51. 9 ± 3.36	40.09 ± 6.26^##^	19.33 ± 9.71^#^
KK-Ay + BSHX 2 g/kg	105.8 ± 15.53	74 ± 8.54	64.93 ± 10.21	59.87 ± 5.37	45.33 ± 5.67	29.54 ± 18.15
KK-Ay + Met 250 mg/kg	102.3 ± 8.62	79.4 ± 4.12	64 ± 6.57	51.74 ± 4.89	46.33 ± 3.51	41.67 ± 7.64

Data are expressed as mean ± SD (*n* = 10). Time, seconds. ^*∗∗*^*P* < 0.01 vs. the C57BL/6J group. ^#^*P* < 0.05, ^##^*P* < 0.01 vs. the KK-Ay group.

## Data Availability

The datasets used and analyzed during the current study are available from the corresponding author upon request.

## Abbreviations

AGEs: Advanced glycation end products
BSHX: Bushen Huoxue prescription
COX-2: Cyclooxygenase-2
FBG: Fasting blood glucose
FINS: Fasting serum insulin
HDL-C: High-density lipoprotein cholesterol
HPLC: High-performance liquid chromatography
IL-6: Interleukin-6
iNOS: Inducible nitric oxide synthase
ITT: Insulin tolerance test
LDL-C: Low-density lipoprotein cholesterol
MAPK: Mitogen-activated protein kinase
MWM: Morris water maze
NF-*κ*B: Nuclear factor-*κ*B
OGTT: Oral glucose tolerance test
PGE2: Prostaglandin E2
P-p38: Phosphorylated p38 mitogen-activated protease
RAGE: Receptors of AGEs
RBG: Random blood glucose
T2DM: Type 2 diabetes mellitus
TC: Total cholesterol
TG: Total triglycerides
TNF-*α*: Tumor necrosis factor-*α*.

## Supplementary Materials

Supplementary Materials Supplementary data file includes the main components and sources of BSHX Prescription.
