# Supplementary material for: Effect of Bushen Huoxue Prescription on Cognitive Dysfunction of KK-Ay Type 2 Diabetic Mice
Source: Evid Based Complement Alternat Med. 2021 Mar 12;2021:6656362. doi: 10.1155/2021/6656362 (PMC7981179; doi:10.1155/2021/6656362)
Supplement: Supplementary Materials — Supplementary data file includes the main components and sources of BSHX Prescription. [file 6656362.f1.doc]

**The main components and sources of BSHX Prescription**

| **No. ^#^** | **Rt (min)** | **Identification** | **Source attribution*** | | | | | | |
| --- | --- | --- | --- | --- | --- | --- | --- | --- | --- |
|  |  |  | Y | F | C | W | T | G | S |
| **F1** | 48.19 | Limocitrol-*O*-glucoside | + |  |  |  |  |  |  |
| **F2** | 55.81 | Limocitrol-*O*-glucoside | + |  |  |  |  |  |  |
| **F3** | 58.79 | Limocitrol-*O*-glucoside | + |  |  |  |  |  |  |
| **F4** | 69.17 | Quercetin-*di*-*O*-glucoside | + | + | + |  | + | + |  |
| **F5** | 71.12 | Quercetin-*di*-*O*-glucoside | + | + | + |  | + | + |  |
| **F6** | 71.65 | Quercetin-*O*-rhamonosyl-*di*-*O*-glucoside | + | + | + |  | + | + |  |
| **F7** | 71.85 | Dihydrokaempferol-*O*-glucoside | + | + | + |  | + | + |  |
| **F8** | 76.35 | Kaempferol-*di*-*O*-glucoside | + | + | + |  | + | + |  |
| **F9** | 88.92 | Quercetin-*O*-glucosyl-*O*-xyloside | + | + | + |  | + | + |  |
| **F10** | 89.67 | Quercetin-*O*-glucosyl-*O*-xyloside | + |  |  |  |  |  |  |
| **F11** | 90.32 | Yinyanghuo D-*O*-glucoside | + |  |  |  |  |  |  |
| **F12** | 91.43 | Kaempferol-*di*-*O*-glucoside | + | + | + |  | + | + |  |

| **No. ^#^** | **Rt (min)** | **Identification** | **Source attribution*** | | | | | | |
| --- | --- | --- | --- | --- | --- | --- | --- | --- | --- |
|  |  |  | Y | F | C | W | T | G | S |
| **F13** | 91.94 | Quercetin-*O*-glucoside | + | + | + |  | + | + | + |
| **F14** | 93.06 | Quercetin-*O*-glucoside | + | + | + |  | + | + | + |
| **F15** | 95.39 | Kaempferol-*O*-rutinoside | + | + | + |  | + | + |  |
| **F16** | 98.32 | Kaempferol-*O*-rutinoside | + | + | + |  | + | + |  |
| **F17** | 100.7 | Kaempferol-*O*-glucoside | + | + | + |  | + | + |  |
| **F18** | 102.5 | Dihydrokaempferol-*O*-methyl-*O*-glucuronide | + |  | + |  | + | + |  |
| **F20** | 107.7 | Desmethylicaritin-*O*-rhamnosyl-*O*-glucoside | + |  |  |  |  |  |  |
| **F21** | 108.8 | Epimedoside E | + |  |  |  |  |  |  |
| **F22** | 109.8 | Desmethylicaritin-*O*-rhamnosyl | + |  |  |  |  |  |  |
|  |  | -*O*-glucuronide |  |  |  |  |  |  |  |
| **F23** | 109.9 | Icaritin-*O*-rutinoside | + |  |  |  |  |  |  |
| **F24** | 110.1 | 1-​Propanone*,* 1-​[2-​(*O*-glucopyrano syloxy)​-​6-​hydroxy-​4-​methoxy phenyl]​-​3-​(4-​methoxyphenyl)​- | + | + | + |  | + | + |  |
| **F25** | 111 | Sagittasine C | + |  |  |  |  |  |  |
| **F26** | 113.4 | Icaritin-*O*-rutinoside | + |  |  |  |  |  |  |
| **F27** | 114.8 | Methylepimediside A | + |  |  |  |  |  |  |
| **F28** | 114.9 | Icaritin-*O*-glucosyl-  *O*-rutinoside | + |  |  |  |  |  |  |
| **F29** | 115 | Wushanicariin | + |  |  |  |  |  |  |
| **F30** | 115.6 | Epimedin B | + |  |  |  |  |  |  |
| **F31** | 115.6 | Icaritin-*tri*-*O*-glucoside | + |  |  |  |  |  |  |
| **F32** | 116.2 | Icaritin-*O*-rhamonosyl-  *O*-rutinoside | + |  |  |  |  |  |  |
| **F33** | 116.7 | Icaritin-*O*-rhamonosyl-  *O*-rutinoside | + |  |  |  |  |  |  |

| **No. ^#^** | **Rt (min)** | **Identification** | **Source attribution*** | | | | | | |
| --- | --- | --- | --- | --- | --- | --- | --- | --- | --- |
|  |  |  | Y | F | C | W | T | G | S |
| **F34** | 117.1 | Epimediside A/Icariine | + |  |  |  |  |  |  |
| **F34** | 117.4 | Epimediside A/Icariine | + |  |  |  |  |  |  |
| **F35** | 118.5 | Epimedokoreanoside II/  Sagittatoside C | + |  |  |  |  |  |  |
| **F36** | 118.6 | 8-Dihydroprenylquercetin-*O*  -methyl-*tri*-*O*-acetyl-*O*-  rutinoside | + |  |  |  |  |  |  |
| **F37** | 120.8 | 8-Dihydroprenylquercetin-  *tri*-*O*-acetyl-*O*-rutinoside | + |  |  |  |  |  |  |
| **F38** | 121.7 | Icaritin-*di*-*O*-acetyl-*O*-glucosyl-*O*-rutinoside | + |  |  |  |  |  |  |
| **F38** | 121.8 | Icaritin-*di*-*O*-acetyl-*O*-glucosyl | + |  |  |  |  |  |  |
|  |  | -*O*-rutinoside |  |  |  |  |  |  |  |
| **F39** | 124.7 | 8-Dihydroprenylquercetin-*di*-  *O*- cinnamoyl -*O*-glucoside | + |  |  |  |  |  |  |
| **F40** | 131.3 | Epimedin K | + |  |  |  |  |  |  |
| **F41** | 131.7 | Icaritin-*tri*-*O*-acetyl-*O*-rutinoside | + |  |  |  |  |  |  |
| **F42** | 133.2 | Icaritin-*tri*-*O*-methyl-*O*-rutinoside | + |  |  |  |  |  |  |
| **F43** | 133.2 | Epimedin K | + |  |  |  |  |  |  |

| **No. ^#^** | **Rt**  **(min)** | **Identification** | **Source attribution*** | | | | | | |
| --- | --- | --- | --- | --- | --- | --- | --- | --- | --- |
|  |  |  | Y | F | C | W | T | G | S |
| **P1** | 3.68 | Caffeic acid-*di*-*O*-acetyl-*O*-  glucosyl-*O*-glucuronide |  |  |  | + | + |  |  |
| **P2** | 32.24 | Amabiloside |  |  |  | + |  |  |  |
| **P3** | 41.78 | Ellagic acid-*O*-xylopyranoside |  | + |  |  |  |  |  |
| **P4** | 47.99 | Quinic acid-*O*-caffeoyl-*O*-glucoside |  |  | + |  |  |  |  |
| **P5** | 48.25 | Quinic-*O*-caffeoyl-*O*-glucoside |  |  | + |  |  |  |  |
| **P6** | 50.59 | Caffeoylquinic acid |  |  | + |  |  |  |  |
| **P7** | 51.14 | 1*,* ​2*,* 3-​Benzenetriol*,* 4-​(2-  hydroxy ethenyl)-*O*-  xylosyl-*O*-glucoside |  |  |  | + |  |  |  |
| **P8** | 52.38 | Quinic acid-*O*-caffeoyl-*O*-glucoside |  |  | + |  |  |  |  |
| **P9** | 55.4 | Caffeic acid-*O*-glucoside |  |  | + |  | + |  |  |
| **P10** | 55.55 | Quinic acid-*O*-caffeoyl-*O*-glucoside |  |  | + |  |  |  | + |
| **P11** | 56.16 | Caffeic acid-*O*-glucoside |  |  | + |  | + |  |  |
| **P12** | 58.7 | Cinnamoylquinic acid |  |  | + |  |  |  |  |
| **P13** | 58.74 | Quinic acid-*O*-caffeoyl-*O*-glucoside |  |  | + |  |  |  |  |
| **P14** | 58.99 | Ellagic acid-*O*-xylopyranoside |  | + |  |  |  |  |  |
| **P15** | 60.86 | Caffeic acid-*O*-glucoside |  |  | + |  | + |  |  |
| **P16** | 61.42 | Quinic acid-*O*-caffeoyl-*O*-glucoside |  |  | + |  |  |  |  |

| **No. ^#^** | **Rt**  **(min)** | **Identification** | **Source attribution*** | | | | | | |
| --- | --- | --- | --- | --- | --- | --- | --- | --- | --- |
|  |  |  | Y | F | C | W | T | G | S |
| **P17** | 61.46 | Caffeic acid-*O*-glucoside |  |  | + |  | + |  |  |
| **P18** | 62.55 | Ellagic acid 4-*O*-xyloside |  | + |  |  |  |  |  |
| **P19** | 62.61 | 4-((1E)-3-methoxy-3-oxoprop-1  -en-1-yl)phenyl (2E)-3-(3*,* 4-dihydroxyphenyl)-2-propenoate |  |  | + |  |  | + |  |
| **P20** | 63.6 | Quinic acid-*O*-caffeoyl-*O*-glucoside |  |  | + |  |  |  |  |
| **P21** | 64.17 | Caffeoylquinic acid |  |  | + |  |  |  |  |
| **P21** | 64.17 | Caffeoylquinic acid |  |  | + |  |  |  |  |
| **P22** | 65.14 | Caffeoylquinic acid |  |  | + |  |  |  |  |
| **P23** | 68.14 | Cinnamoylquinic acid |  |  | + |  |  |  |  |
| **P24** | 72.91 | Cinnamoylquinic acid |  |  | + |  |  |  |  |
| **P25** | 73.11 | Cinnamoylquinic acid |  |  | + |  |  |  |  |
| **P26** | 74.12 | Cinnamoylquinic acid |  |  | + |  |  |  |  |
| **P27** | 78.67 | Cinnamoylquinic acid |  |  | + |  |  |  |  |
| **P28** | 106.1 | Quinic acid-*tri*-*O*-glucoside |  |  | + |  |  |  |  |

| **No. ^#^** | **Rt (min)** | **Identification** | **Source attribution*** | | | | | | |
| --- | --- | --- | --- | --- | --- | --- | --- | --- | --- |
|  |  |  | Y | F | C | W | T | G | S |
| **A1** | 2.968 | Adenosine |  |  | + |  | + |  |  |
| **A2** | 3.292 | 2-​Propenoic acid, 3-​phenyl-​  2-​[(2-​phenylacetyl)​amino]​- |  |  | + |  | + |  |  |
| **A3** | 25 | Guanosine |  |  | + |  | + |  |  |
| **A4** | 60.07 | N1-dihydrocaffeoyl-N3-  dihydro caffeoylspermidine-  *di*-*O*-glucoside |  |  |  |  | + |  |  |
| **A5** | 61.06 | N1-dihydrocaffeoyl-N3-  dihydro caffeoylspermidine-  *di*-*O*-glucoside |  |  |  |  | + |  |  |
| **A6** | 62.5 | N1-caffeoyl-N3-dihydrocaffeoyl spermidine-*O*-glucoside |  |  |  |  | + |  |  |
| **A7** | 63.91 | N1-caffeoyl-N3-dihydrocaffeoyl spermidine-*di*-*O*-glucoside |  |  |  |  | + |  |  |
| **A8** | 63.96 | N1-caffeoyl-N3-dihydrocaffeoyl spermidine-*O*-glucoside |  |  |  |  |  | + |  |
| **A9** | 65.7 | N1-dihydrocaffeoyl-N3-dihydro caffeoylspermidine |  |  |  |  |  | + |  |
| **A10** | 66.19 | N1-caffeoyl-N3-dihydrocaffeoyl spermidine-*O*-glucoside |  |  |  |  |  | + |  |
| **A10** | 66.24 | N1-caffeoyl-N3-dihydrocaffeoyl spermidine-*O*-glucoside |  |  |  |  |  | + |  |
| **A11** | 67.03 | N1-caffeoyl-N3-dihydrocaffeoyl spermidine-*O*-glucoside |  |  |  |  |  | + |  |
| **A12** | 67.82 | N1-caffeoyl-N3-dihydrocaffeoyl spermidine-*O*-glucoside |  |  |  |  |  | + |  |
| **A13** | 70.02 | N1-caffeoyl-N3-dihydrocaffeoyl spermidine |  |  |  |  |  | + |  |
| **No. ^#^** | **Rt (min)** | **Identification** | **Source attribution*** | | | | | | |
|  |  |  | Y | F | C | W | T | G | S |
| **A14** | 70.57 | 2-​Propenamide, 3-​(3*,* ​4-​dimethoxy phenyl)​-​N-​[2-​(4-​methoxyphenyl)​ethyl]​*-​,* (2E)​- |  |  |  |  |  | + |  |
| **A15** | 86.94 | Benzenepropanamide, N-​[3-​[[4-​[(3-​aminopropyl)​amino]​butyl]​amino]^-^propyl]-​3*,* ​4-​dihydroxy- |  |  |  |  |  | + |  |
| **A16** | 92.55 | Benzenepropanamide, N-​[3-​[[4-​[(3-​aminopropyl)​amino]butyl]​amino]^-^propyl]^-^-​3*,* ​4-​dihydroxy- |  |  |  |  |  | + |  |
| **A17** | 97.55 | Benzenepropanamide, N-​[3-​[[4-​[(3-​aminopropyl)​amino]^-^​butyl]^-^​amino]^-^​propyl]^-^​-​3*,* ​4-​dihydroxy- |  |  |  |  |  | + |  |
| **G1** | 3.405 | Mannopyranose-*O*-mannopyranosyl |  |  |  |  |  | + |  |
| **G2** | 3.462 | ​Mannopyranose-*O*-​mannopyranosyl -​(1→3)​-​*O*-mannopyranosyl-​(1→2)​- |  |  |  |  |  | + |  |
| **G3** | 3.462 | 2-α-Nigerosylglucose |  |  |  |  |  | + |  |
| **G4** | 4.478 | L-glycero-*O*-galacto heptitol-3*,*6-anhydro-*O*-glucoside |  |  | + |  |  |  |  |
| **G5** | 4.737 | L-ascorbic acid-*O*-glucuronide |  |  |  |  |  | + |  |
| **G6** | 5.253 | 2-α-Nigerosylglucose |  |  |  |  |  | + |  |
| **G7** | 7.415 | L-Ascorbic acid-*O*-glucoside |  |  | + |  |  |  |  |
| **G8** | 8.48 | L-Ascorbic acid-*O*-glucoside |  |  | + |  |  |  |  |
| **G9** | 11.99 | Erythro-pentofuranuronic acid, methyl ester-2-deoxy-*O*-glucosyl-*O*-glucuronide |  |  |  |  |  | + |  |
| **No. ^#^** | **Rt (min)** | **Identification** | **Source attribution*** | | | | | | |
|  |  |  | Y | F | C | W | T | G | S |
| **G10** | 92.6 | Acteoside |  |  |  |  |  | + |  |
| **G11** | 97.3 | Acteoside |  |  |  |  |  | + |  |
| **FA1** | 106.4 | Pentadecenoic acid, 15-hydroxy-*O*-glucoside |  | + |  |  | + |  |  |
| **FA2** | 106.4 | Pentadecenoic acid, 15-hydroxy-*O*-glucoside |  | + |  |  | + |  |  |
| **FA3** | 112.6 | Pentadecenoic acid, 15-hydroxy-*O*-glucoside |  | + |  |  | + |  |  |
| **FA4** | 114.4 | Pentadecenoic acid, 15-hydroxy-*O*-glucoside |  | + |  |  | + |  |  |
| **FA5** | 116.5 | Lyciumoside VIII |  |  |  |  |  | + |  |
| **FA6** | 119.7 | 2, 6, 10, 15-Hexadecatetraene-1, 4, 14-triol, 2, 6, 10, 14-tetramethyl-*O*-glucoside |  |  |  |  |  | + |  |
| **FA7** | 121.4 | 2, 6, 10, 15-Hexadecatetraene-1, 4, 14-triol, 2, 6, 10, 14-tetramethyl-*O*-glucoside |  |  |  |  |  | + |  |
| **FA8** | 122.8 | 2, 6, 10, 15-Hexadecatetraene-1*,* 4, 14-triol, 2, 6, 10, 14-tetramethyl-*O*-glucoside |  |  |  |  |  | + |  |

| **No. ^#^** | **Rt (min)** | **Identification** | **Source attribution*** | | | | | | |
| --- | --- | --- | --- | --- | --- | --- | --- | --- | --- |
|  |  |  | Y | F | C | W | T | G | S |
| **L1** | 75.02 | Dibenzo[a*,*c]^-^cyclooctene-1, 2, 6, 11, 12-pentol, 5,6,7,8-tetrahydro-3,10-dimethoxy-6*,*7-dimethyl-*O*-glucoside |  |  |  | + |  |  |  |
| **L2** | 96.04 | Epipinoresinol-*O*-glucoside |  |  |  | + | + | + | + |
| **L3** | 112.7 | Hydroxyisocupressoside B |  |  |  | + | + | + |  |
| **L4** | 145.2 | Isoschizandrin |  |  |  | + |  |  |  |
| **T1** | 46.83 | Geniposidic acid |  |  | + |  |  |  |  |
| **T2** | 90.54 | Cyclopenta pyran-4-carboxylic acid-acetyl-1,4,5,7-tetrahydro-1-hydroxy-methyl ester-*O*-glucoside |  |  | + |  |  |  |  |
| **S1** | 87.08 | 9, 19-Cyclolanosta-22, 24-dien-26-oic acid, 3-oxo-, (22Z, 24E)- |  | + |  |  |  |  |  |

*Y: 淫羊藿（*Epimedium brevicornu* Maxim. ）

F: 覆盆子（*Rubus chingii* Hu. ）

C: 车前子（*Plantago asiatica* L. ）

T: 菟丝子（*Cuscuta chinensis* Lam.）

G: 枸杞子（*Lycium barbarum* L ）

W：五味子（*Schizandra chinensis (Turcz.)* Baill.）

S：水蛭 （*Hirudo nipponica* Whitman）
